# Strawberry cryptochrome FvCRY1 and FvCRY2 transcriptionally regulate anthocyanin biosynthesis and sugar metabolism

**DOI:** 10.1186/s43897-025-00197-5

**Published:** 2025-12-02

**Authors:** Lianxi Zhang, Anqi Lin, Xiaoyi Bi, Yuxuan Zhu, Lin Ye, Jiwen Lian, Pengbo Xu, Hongli Lian

**Affiliations:** Shanghai Collaborative Innovation Center of Agri-Seeds, School of Agriculture and Biology, Shanghai Jiao Tong University, Shanghai, China

**Keywords:** Strawberry, Cryptochrome, Anthocyanin, Sugar, Transcriptional regulation

## Abstract

Anthocyanin pigmentation and soluble sugar content are important factors affecting the quality of strawberry fruits, and their accumulation processes are profoundly influenced by light signals. However, the molecular mechanism through which light regulates this process remains to be fully elucidated. Here, we identified two blue light receptors, FvCRY1 and FvCRY2, from woodland strawberry. Functional complementation in *Arabidopsis* cryptochrome mutant confirmed their photoperceptor roles. Stable overexpression of FvCRYs in cultivated strawberry (*Fragaria* × *ananassa* 'Ningyu') significantly enhanced anthocyanin accumulation and soluble sugar content, concomitant with upregulation of key biosynthetic genes. Strikingly, yeast one-hybrid and dual-luciferase assays revealed FvCRYs possess intrinsic transcriptional activation domains that directly bind E-box cis-elements in promoters of anthocyanin synthesis gene *FvCHS2, FvDFR2, FvCHI*, and sugar metabolism-related genes *FvSFP9* and *FvINV*. This DNA-binding capacity is potentiated by blue light, enabling transcriptional expression of anthocyanin synthesis and sugar metabolism genes. Our findings unveil a non-canonical cryptochrome signaling mechanism involving direct receptor-promoter interaction, redefining our understanding of photoreceptor-mediated frui quality regulation.

**Supplementary Information:**

The online version contains supplementary material available at 10.1186/s43897-025-00197-5.

## Core

This study identifies two blue light receptors, FvCRY1 and FvCRY2, in woodland strawberry (*Fragaria vesca*). Functional characterization demonstrates their intrinsic transcriptional activation capacity enhanced by blue light. Crucially, they directly bind to the promoters of key anthocyanin biosynthesis genes (*FvCHS2, FvDFR2, FvCHI*) and sugar metabolism-related genes (*FvSFP9, FvINV*), activating their transcription. Our findings establish a novel regulatory paradigm where FvCRY1 and FvCRY2 regulate both anthocyanin and sugar metabolism during strawberry fruit maturation through directly binding and activating target genes.

## Gene & accession numbers

Information for the genes (*FvCRY1, FvCRY2, FvDFR2, FvCHS2, FvCHI, FvINV, FvSFP9*) discussed in this article is available in the Genome Database for Rosaceae (https://www.rosaceae.org/) under the following accession numbers: *FvCRY1* (FvH4_5g37290), *FvCRY2* (FvH4_4g03570), *FvDFR2* (FvH4_2g39520), *FvCHS2* (FvH4_7g01160), *FvCHI* (FvH4_7g20870), *FvINV* (FvH4_7g27630), and *FvSFP9* (FvH4_4g16900).

## Introduction

Cultivated strawberry (*Fragaria* × *ananassa*), a globally cherished fruit crop in the Rosaceae family, is favored by consumers for its bright color, juicy texture, and rich nutritional value (Liu et al. 2023). Anthocyanins in strawberry fruits, representative plant flavonoid compounds, serve multiple botanical functions, including attracting pollinators and helping plants resist biotic or abiotic stresses (Landi et al. 2015; Wang et al. 2020; LaFountain and Yuan 2021). For human health, flavonoid compounds have multiple functions such as antioxidant, antibacterial, anticancer, and anti-cardiovascular disease functions (Teow et al. 2007; Zhang et al. 2009; Zhao et al. 2013). The diversity of strawberry fruit color is mainly determined by the types and contents of anthocyanins. Existing studies have shown that anthocyanins are catalyzed from phenylalanine as a direct precursor through a series of enzymes including phenylalanine ammonia lyase (PAL), anthocyanidin reductase (ANR), UDP-glucose flavonoid 3-O-glucosyltransferase (UFGT), chalcone synthase (CHS), chalcone isomerase (CHI), flavanone-3-hydroxylase (F3H), dihydroflavonol-4-reductase (DFR), anthocyanidin synthase (ANS) (Welch et al. 2008).

Besides anthocyanin substances, the sugar content in strawberry fruits serves as a crucial quality—evaluating indicator (Liu et al. 2020). Sugar is not merely the fundamental energy source and signaling molecule in organisms but also participates in osmotic homeostasis and numerous other biological processes (Rolland et al. 2002). During sugar accumulation, transporters such as sucrose transporters (SUTs) (Kühn and Grof 2010; Durand et al. 2018), monosaccharide transporters(MSTs) (Johnson and Thomas 2007), sugar facilitator proteins (SFPs) (Poschet et al. 2011), and sugars will eventually be exported transporters (SWEETs) (Chen et al. 2010)play vital roles. For instance, the heterologous expression of apple *MdSUT4.1* in strawberries led to a significant increase in the fruit's sucrose content (Peng et al. 2020), while the silencing of *FaSUT1* in strawberries decreased the sucrose content (Jia et al. 2013). The SFP family gene *AtERDL6*, when mutated or over—expressed in *Arabidopsis*, causes a significant change in leaf sugar content (Poschet et al. 2011). Similarly, the transgenic overexpression of *MdERDL6-1* in apples is associated with an increase in fructose, glucose, and sucrose levels (Zhu et al. 2024). Additionally, FvSFP9 and FvSFP10 have been recently reported to be involved in sugar accumulation in strawberries (Liu et al. 2020).

Sucrose (Suc), the main photosynthetic end—product, undergoes bidirectional conversion via coordinated enzymatic regulation. In the cytosol, invertase (INV) and sucrose synthase (SUS) catabolize sucrose into glucose, fructose, and uridine diphosphate glucose (UDPG). Conversely, these monosaccharides can be reversibly converted back into sucrose through the catalytic action of sucrose phosphate synthase (SPS), with the assistance of energy—dependent phosphorylation reactions. This enzymatic reciprocity creates a dynamic equilibrium between sucrose synthesis and degradation, which is essential for maintaining carbohydrate homeostasis in plant cells (Chen et al. 2022). In citrus, the transient virus induced gene silencing (VIGS) of *CitSUS5* led to a significant reduction in sugar content (Fang et al. 2023). In transgenic apple calli, the silencing of *MdNINV6* caused a significant increase in sucrose content and a significant decrease in glucose and fructose content (Zhang et al. 2024). In strawberries, FaMYB44.2 negatively regulates the accumulation of soluble sugars by inhibiting the expression of *FaSPS3 *(Wei et al. 2018)*.*Moreover, the NAC transcription factors FvRIF/FaRIF are involved in regulating fruit sugar accumulation by modulating the expression of *FaSPS1* and *FaSUS *(Martín-Pizarro et al. 2021; Li et al. 2023)*.*

Light signaling plays a dual role in fruit quality formation: it energizes photosynthesis for carbohydrate synthesis and directly regulates metabolic pathways through photoreceptors (Zoratti et al. 2014; Liu et al. 2018b). Among these, cryptochromes (CRYs), blue light receptors containing a conserved N-terminal photolyase homology region (PHR) and a C-terminal extension (CCE), are pivotal in mediating light-dependent development (Wang and Lin 2020). In *Arabidopsis thaliana*, cryptochromes (CRYs) can regulate photomorphogenesis, flowering, and flavonoid biosynthesis not only by directly interacting with transcription factors such as PIF, CIB1, and ARF (Pedmale et al. 2016), but also by indirectly regulating the protein abundances of transcription factors like HY5 (Saijo et al. 2003) and MYB (Xie et al. 2023). In tomatoes, overexpression of *CRY2* can increase the flavonoid and lycopene contents in fruits (Giliberto et al. 2005), while overexpression of *CRY1a* increases the contents of glucose, fructose, and citric acid and reduces the contents of sucrose and malic acid (Liu et al. 2018a). Recent studies have shown that FaCRY1 can bind to the promoter of ABA catabolism gene *FaCYP707A4* and recruit FaAGO4, a key protein involved in RNA‐directed DNA methylation, to promote ripening via epigenetic regulation of ABA metabolism (Sun et al. 2024). Notably, dark-treated strawberries fail to accumulate anthocyanins and sugars, underscoring light’s indispensability (Xu et al. 2018). Yet, the mechanism by which CRYs directly link light perception to fruit quality remains elusive.

Here, we identify FvCRY1 and FvCRY2 as key blue light receptors in woodland strawberry. Through functional complementation and molecular analyses, we demonstrate that FvCRYs directly bind to the promoters of anthocyanin biosynthesis genes (*FvCHS2, FvDFR2, FvCHI*) and sugar metabolism genes (*FvSFP9, FvINV*), independent of light conditions. Crucially, their transcriptional activation capacity toward these target genes is significantly potentiated by blue light, revealing a two-layered regulatory mechanism: light-independent DNA binding coupled with light-triggered transcriptional activation. This discovery establishes a non-canonical CRY signaling pathway, redefining how photoreceptors directly orchestrate fruit quality.

## Results

### Identification and characterization of blue light receptors FvCRY1 and FvCRY2 in *Fragaria vesca*

**Fig. 1:**
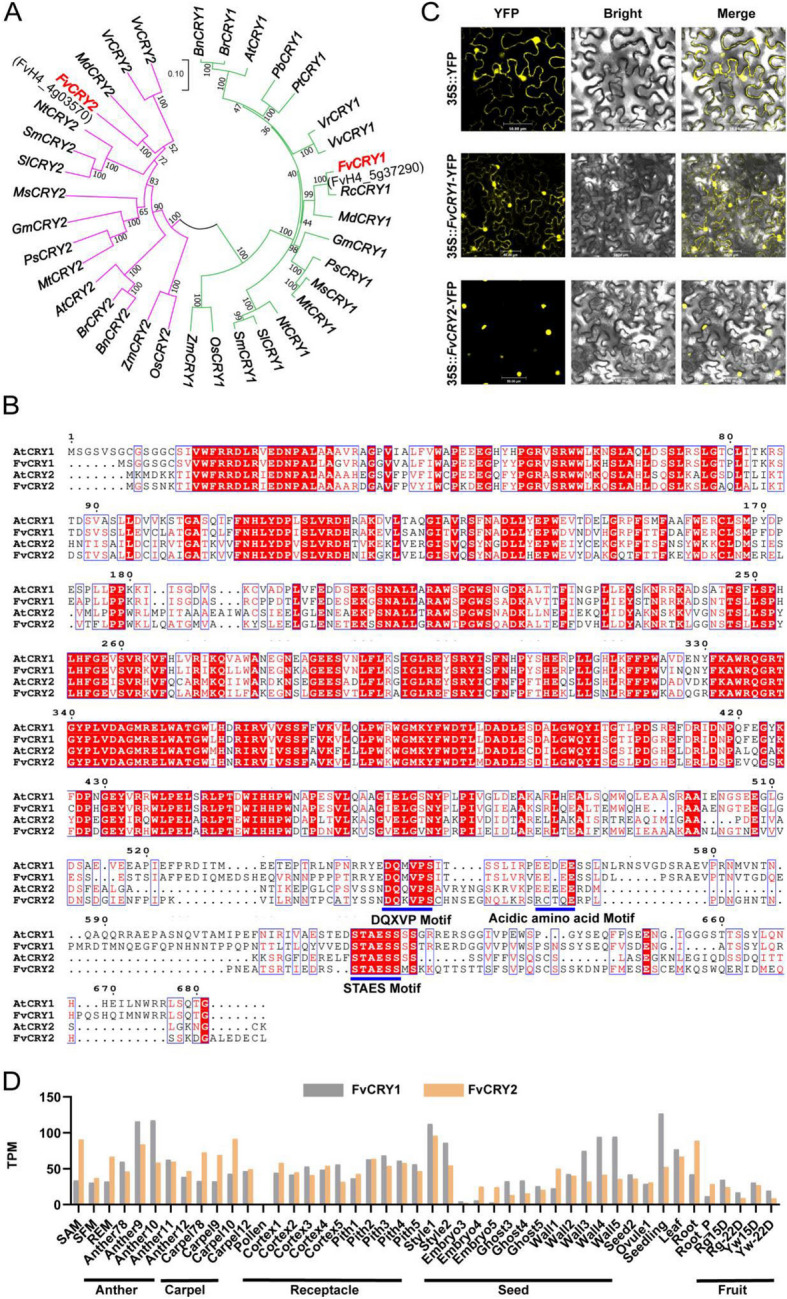
Bioinformatics and expression characteristic of FvCRYs. **A** Phylogenetic analysis of CRYs in strawberry and other species. At, *Arabidopsis thaliana.* Bn, *Brassica napus*. Br, *Brassica rapa*. Fv, *Fragaria vesca*. Gm, *Glycine max*. Md, *Malus domestica*. Ms, *Medicago sativa*. Mt, *Medicago truncatula*. Nt, *Nicotiana tabacum*. Ps, *Pisum sativum*. Pb, *Populus balsamifera*. Pt, *Populus tremula*. Rc, *Rosa chinensis*. Sl, *Solanum lycopersicum*. Sm, *Solanum melongena*. Vr, *Vitis riparia*. Vv, *Vitis vinifera*. Os, *Oryza sativa*. Zm, *Zea mays*. **B** Comparison of protein sequences of FvCRYs and AtCRYs. The blue horizontal lines indicate DAS structural domains. **C** Subcellular localization of FvCRYs. Scale bars = 50 μm. **D** Expression levels (Transcript per Kilobase per Million mapped reads, TPMs) of the *FvCRYs* genes in *F. vesca* from eFP database

To identify the blue light receptors of *Fragaria vesca* (woodland strawberry), we conducted a BLASTp search using the protein sequences of AtCRY1 and AtCRY2 from *Arabidopsis thaliana* against the protein sequences of *Fragaria vesca*. This search determined that two proteins, FvH4_4g03570 and FvH4_5g37290, might be the homologs of AtCRYs. Subsequently, we constructed phylogenetic trees using the CRY1 protein sequences of 19 species and the CRY2 protein sequences of 16 species together with FvH4_4g03570 and FvH4_5g37290 (Fig. 1A). The results showed that FvH4_5g37290 clustered with CRY1 in one group, while FvH4_4g03570 clustered with CRY2 in another group. Based on this, FvH4_5g37290 was named FvCRY1; FvH4_4g03570 was named FvCRY2. Meanwhile, the results of protein sequence alignment showed that FvCRYs exhibited high protein sequence similarity with the AtCRYs in the DNA photolyase domain at the N-terminus (Fig. 1B), similarly, the N-terminus of CRY is conserved across species (Figs. S1, S2). And in the C-terminal region, the markly DAS motifs, including the DQXVP motif, the short acidic amino acid motif, and the STAES motif, were conserved in FvCRYs except that FvCRY2 does not have the short acidic amino acid motif (Fig. 1B).

Afterwards, a *35S* promoter-driven fusion protein expressing FvCRYs and yellow fluorescent protein (YFP) was constructed and transiently expressed in tobacco to investigate the cellular localization of FvCRYs. The results showed that the FvCRY1-YFP signal was detected in the nucleus and cytoplasm of the cell, while the FvCRY2-YFP signal was detected in the nucleus, which are largely consistent with the localization of blue light receptors AtCRY1/AtCRY2 in *Arabidopsis* (Fig. 1C) (Wu and Spalding 2007; Yu et al. 2007).

In addition, querying the strawberry eFP (electronic Fluorescent Pictograph) database (Li et al. 2019), we found that *FvCRY1/FvCRY2* was mainly expressed in anther, carpel, seed, and receptacle tissues, but also to some extent in other tissues, implying that they are widely involved in multiple processes of strawberry growth and development (Fig. 1D).

### Functional validation of FvCRY1 and FvCRY2 as blue light receptors through complementation experiments in *Arabidopsis* cryptochrome mutants

**Fig. 2:**
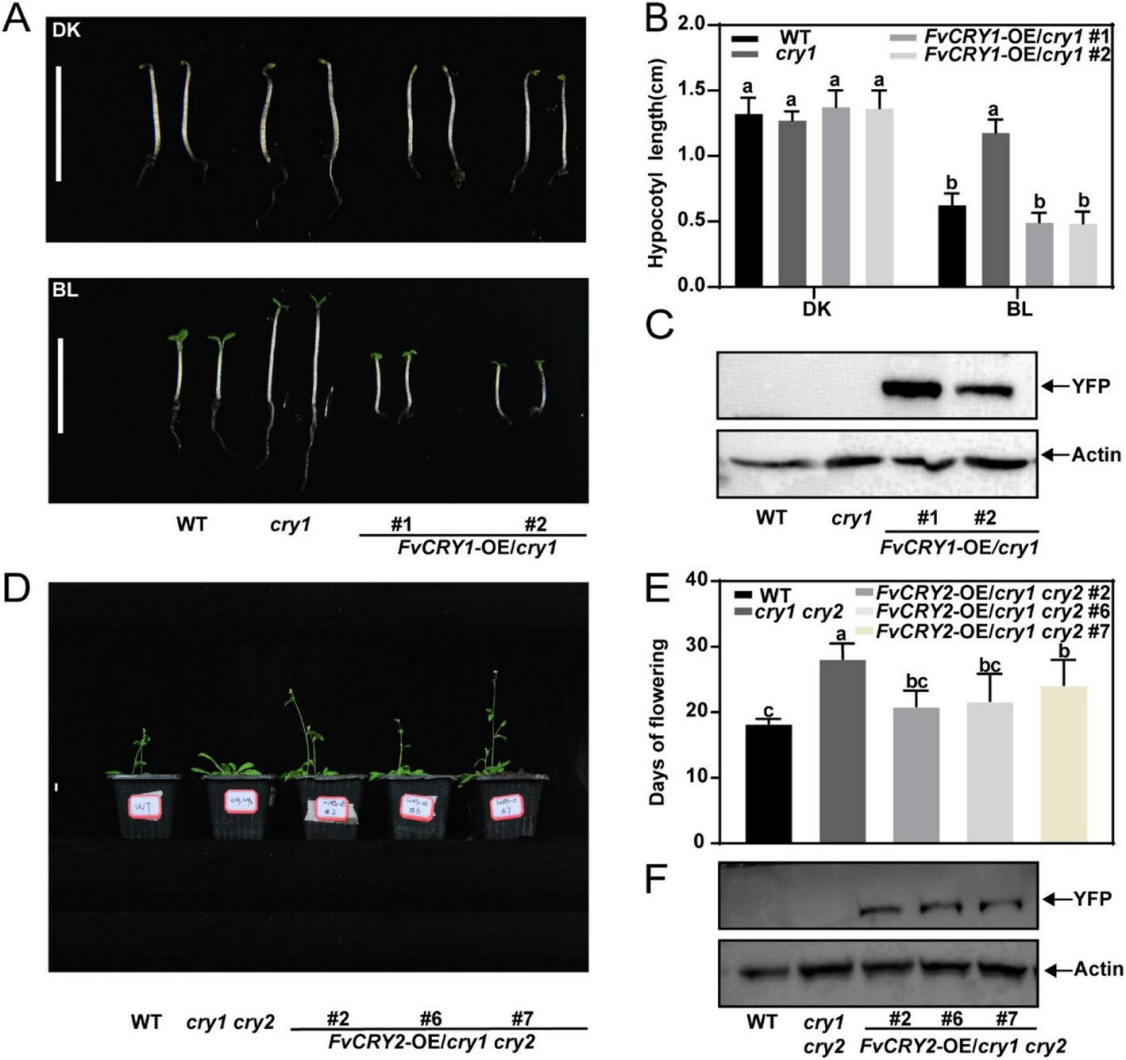
Functional Validation of FvCRY1 and FvCRY2. **A** Hypocotyl phenotypes of WT, *cry1*, and *FvCRY1-*OE*/cry1* under dark and blue light. Scale bars = 1 cm. **B** Determination of hypocotyl length in WT, *cry1*, and *FvCRY1-*OE*/cry1* under dark and blue light treatments. Data are means ± SD (*n* = 20). **C** Detection of FvCRY1-YFP fusion proteins in transgenic *Arabidopsis*. **D** Flowering phenotypes of WT, *cry1 cry2*, *FvCRY2-*OE*/cry1 cry2* under long daylight. Scale bars = 1 cm. **E** Statistics on flowering time of WT, *cry1 cry2*, *FvCRY2-*OE*/cry1 cry2* under long daylight. Data are means ± SD(*n* = 10). **F** Detection of FvCRY2-YFP fusion proteins in transgenic *Arabidopsis*. Different letters indicate significant differences using ANOVA (*P* < 0.05). WT: Wild-Type

We conducted functional complementation experiments by overexpressing *FvCRYs* in the *Arabidopsis cry* mutant to further confirm whether FvCRYs proteins function as blue-light receptors. The hypocotyls of WT, *FvCRY1*-OE/*cry1*, were found to be significantly suppressed under blue-light conditions compared with the mutant *cry1* (Figs. 2A, B). These results indicate that FvCRY1 has the same function as AtCRY1, validating the function of FvCRY1 as a blue light receptor.

CRY2 in *Arabidopsis* mainly functions as a promotive of flowering. In *Arabidopsis cry1 cry2* double mutant, flowering time is significantly delayed under long day light. Transformation of the *Arabidopsis cry1 cry2* double mutant using the *35S* promoter-driven overexpression vector pHB-FvCRY2-YFP revealed that the flowering time of the transgenic plants was significantly earlier and had recovered to the level of the wild type (Figs. 2D, E). These results indicated that FvCRY2 shared the same function with *Arabidopsis* AtCRY2, further validating the function of FvCRY2 as a blue light receptor. Addtionally, western blot results showed that FvCRY1 and FvCRY2 were successfully expressed in *cry1*, *cry1 cry2* backgrounds, respectively (Figs. 2C, F).

### FvCRYs promote the accumulation of anthocyanins and sugars in strawberry

**Fig. 3:**
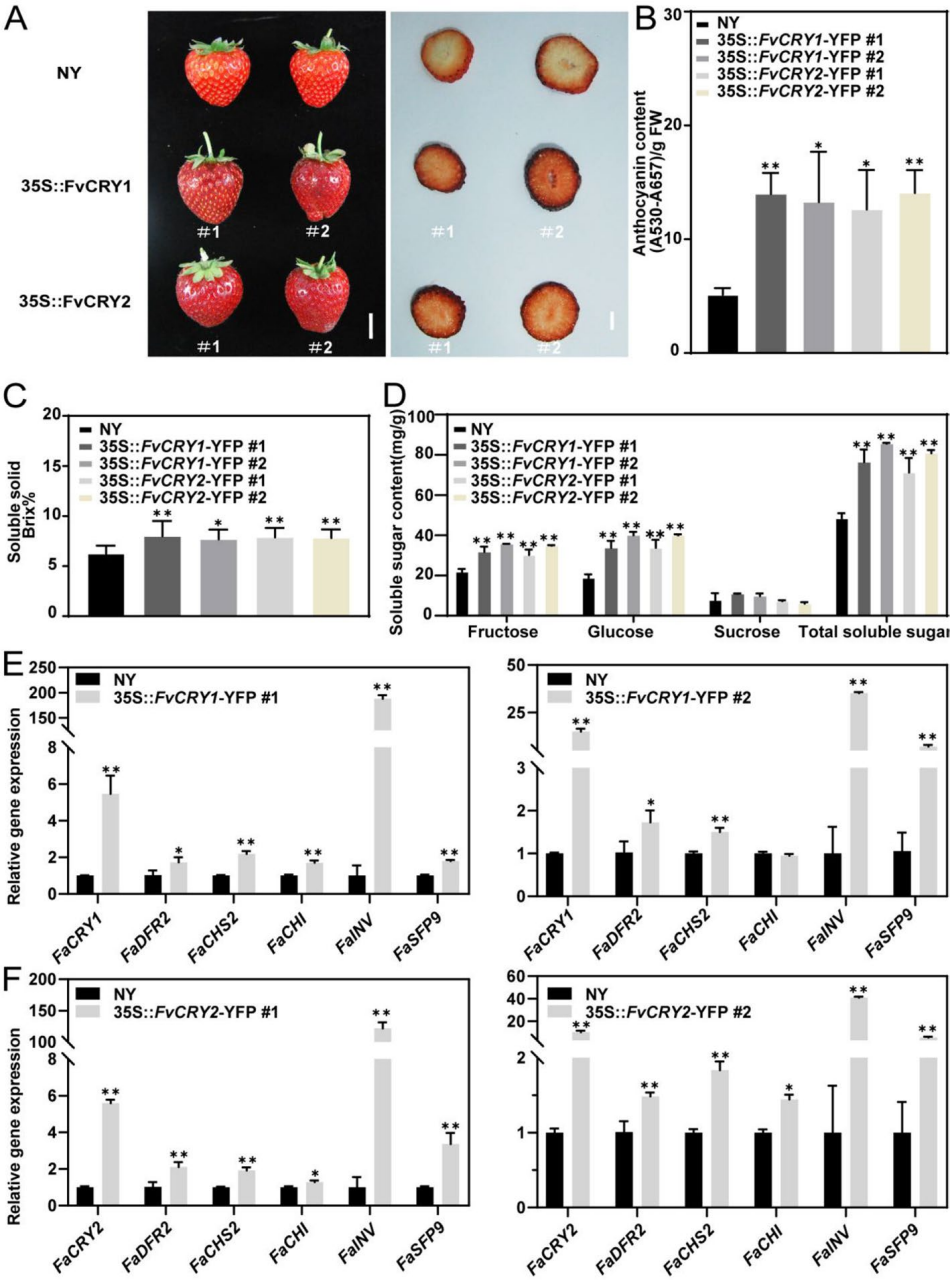
FvCRYs regulate anthocyanin accumulation and sugar metabolism in cultivated strawberries. **A** FvCRYs transgenic strawberry fruit color phenotype. Cultivated strawberry ‘Ningyu’ served as control. Scale bars = 1 cm. **B** Determination of anthocyanin content in the transgenic strawberry. **C** Determination of soluble solids in the transgenic strawberry. **D** Determination of the content of soluble sugar in the transgenic strawberry. **E** Relative expression levels of anthocyanin and sugar metabolism related genes in *FvCRY1* overexpressing strawberry fruits were analyzed by RT-qPCR. **F** Relative expression levels of anthocyanin and sugar metabolism related genes in *FvCRY2* overexpressing strawberry fruits were analyzed by RT-qPCR. Asterisks indicate a significant difference relative to the control (*, *P* < 0.05, **, *P* < 0.01, Student's t-test). Data are means ± SD from three independent biological replicates

Light is a key factor affecting fruit quality. To investigate whether the photoreceptors FvCRYs are involved in the regulation anthocyanin and sugar accumulation, we obtained *FvCRY1* stable overexpression Lines 35S::*FvCRY1—*YFP and *FvCRY2* stable overexpression Lines 35S::*FvCRY2—*YFP in the octoploid cultivated strawberry 'Ningyu'. Compared with the wild type, overexpression of FvCRYs resulted in a significant increase in anthocyanin content and total soluble solids content (Figs. 3A - C, S3) as well as fructose content and glucose content (Fig. 3D). RT-qPCR results showed that overexpression of either FvCRY1 or FvCRY2 significantly increased the expression levels of flavonoid synthesis genes *FvDFR2*, *FvCHS*, *FvCHS2*, and sugar metabolism and transport genes *FvINV* and *FvSFP9* (Figs. 3E, F), which was consistent with anthocyanin accumulation and soluble solids in transgenic strawberries, implying that the FvCRYs may promote strawberry fruit anthocyanin and sugar accumulation by regulating the expression of these quality-related genes.

### Blue light promotes the transcriptional activation ability of FvCRYs

**Fig. 4:**
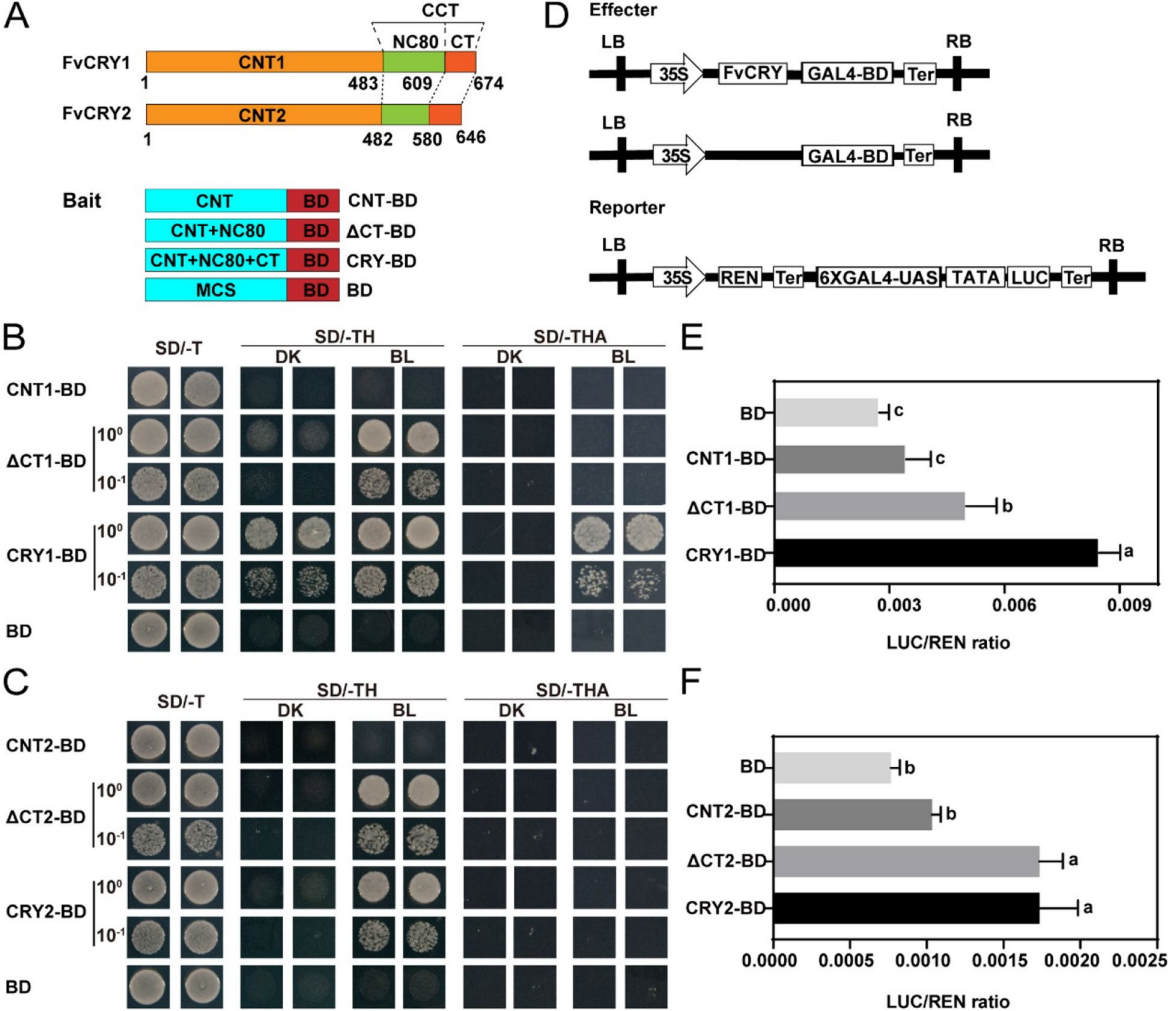
FvCRYs has blue light-promoted transcriptional activation activity. **A** Protein structural domains of FvCRYs and schematic representation of the construction of yeast self-activating experimental vectors. CNT, CRY N-terminal domain; CCT, CRY C-terminal domain; CT, C-terminal tail; NC80, 80 residues between the N- and C-terminal domains; ΔCT indicates the absence of the CT structural domain. **B** and **C** Self-activating activity of FvCRY1 (**B**) or FvCRY2 (**C**) in yeast cells. SD/-T, SD/-TH, SD/-THA stands for different defective media; DK: Dark; BL: Blue Light. **D** Diagram of the effector and reporter constructs used in the dual-luciferase assays. *35S* promoter-driven FvCRYs-GAL4-BD fusion protein was used as effector, *GAL4-UAS-TATA* promoter-driven firefly luciferase (LUC) as reporter and *35S* promoter-driven renilla luciferase (REN) as internal reference. **E** and **F** The ability of FvCRYs-BD fusion proteins of different lengths to activate the *GAL4-UAS-TATA* promoter was investigated by Dual-LUC assay. Different letters indicate significant differences using ANOVA (*P* < 0.05). Data are means ± SD from three independent biological replicates

During the previous FvCRY1 interaction protein screen, we found that FvCRY1 has obvious transcription factor-like self-activation in yeast. Considering its role as a blue light receptor but with transcriptional activation capacity, this prompted us to further explore the transcriptional activation capacity of FvCRY1. Yeast self-activation experiments were performed on different truncated forms of FvCRY1, according to their structural domains (Fig. 4A), and the results showed that blue light significantly promoted the self-activating activity of the full-length FvCRY1 on medium lacking tryptophan and histidine (SD/-TH). When FvCRY1 was missing the CT portion of the C-terminal, it (ΔCT1-BD) exhibited blue light-dependent self-activation activity. On this basis, the truncated FvCRY1 (CNT1-BD) no longer possessed self-activating activity if the C-terminal NC80 structural domain continued to be missing (Fig. 4B). This suggests that the C-terminal NC80 domain is important for the self-activating activity of FvCRY1. However, under more stringent conditions of screening pressure (lack of tryptophan, histidine, and adenine, SD/-THA), we found that the full-length of FvCRY1 also exhibited blue-light-dependent self-activating activity (Fig. 4B).

Similarly, we performed truncation experiments on FvCRY2, and unlike FvCRY1, the full length of FvCRY2 showed blue-light-dependent self-activating activity in medium lacking tryptophan and histidine (SD/-TH), but deletion of NC80 (CNT2-BD) rendered FvCRY2 no longer self-activating (Fig. 4C). Further on medium lacking tryptophan, histidine and adenine (SD/-THA), both full-length FvCRY2 and FvCRY2 deficient in CT (ΔCT2-BD) no longer had self-activating activity (Fig. 4C). The above results suggest that the NC80 structural domain at the C-terminal of FvCRYs is essential for their self-activating activity, and that FvCRY1 has a stronger self-activating ability than FvCRY2. Then, we further verified whether FvCRYs could exert transcriptional activation activity in plants, and the results showed that both full-length and truncated FvCRYs missing the CT region activated the expression of the *LUC* reporter gene compared with the N terminus alone (Figs. 4D-F). It further indicates that FvCRYs have transcriptional activation ability and their transcriptional activation activity requires the involvement of the C-terminus.

### FvCRYs can directly bind and activate the expression of genes related to anthocyanin and sugar accumulation

**Fig. 5:**
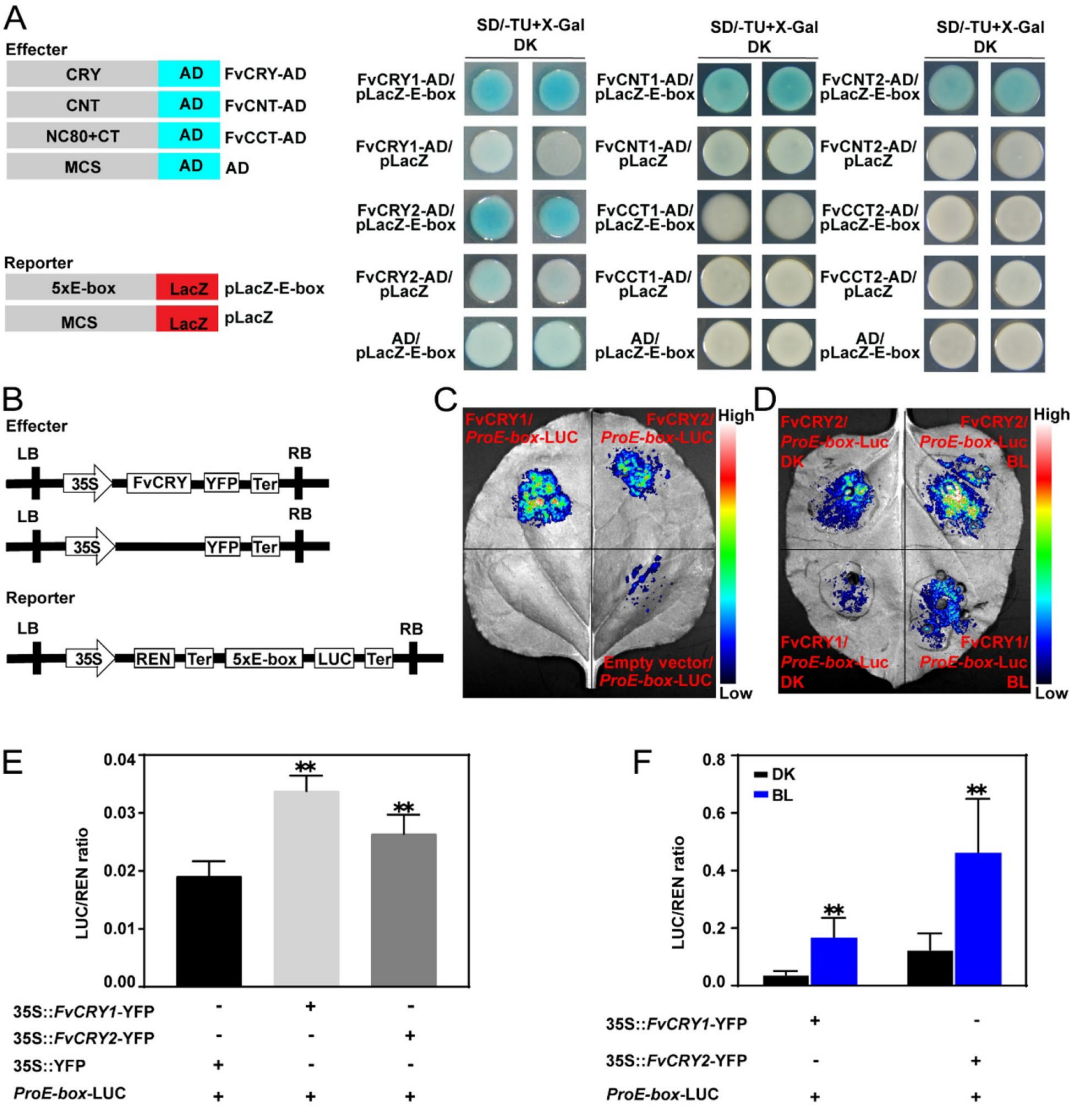
FvCRYs can directly bind the E-box cis-element. **A** Yeast one-hybrid assay showing full-length and N-terminal of FvCRYs bind to the E-box motif. CNT, CRY N-terminal domain; CCT, CRY C-terminal domain; CT, C-terminal tail; NC80, 80 residues between the N- and C-terminal domains; SD/-TU, the medium deficient in tryptophan and uracil; X-Gal, 5-Bromo-4-chloro-3-indolyl β-D-galactopyranoside. **B** Diagram of the effector and reporter constructs used in the Dual-LUC assays. **C** and **D** Dual-LUC imaging assay indicating FvCRYs activate the E-box and blue light significantly enhances the activation of E-box by FvCRYs. **E** and **F** The relative quantification of LUC activity for the Dual-LUC assay in (**C**) and (**D**). The LUC/REN ratio of control was set to 1. Asterisks indicate a significant difference relative to the control ( *, *P* < 0.05, **, *P* < 0.01, Student's *t*-test). Data are means ± SD from three independent biological replicates. DK: Dark; BL: Blue Light

The studies in *Arabidopsis* showed that AtCRY2 could enrich for E-box cis-elements, but it was not clear whether AtCRY2 could directly bind E-box cis-elements (Pedmale et al. 2016). We confirmed by Y1H experiments that FvCRYs are able to bind directly to the E-box. Additionally, this binding occurs at the N-terminus rather than the C-terminus of the FvCRYs (Fig. 5A). Further by dual luciferase assay, we found that FvCRYs could also activate *LUC* expression after binding to E-box cis-elements (Figs. 5C, E), and this activation disappeared after mutating E-box (CACGTGTCAG) to mE-box (CAAAAAAAG) (Fig. S4). Moreover, this activation was more obvious under blue light (Fig. 5D, F). These results imply that FvCRYs may function by directly binding to and activating the expression of downstream target genes.

**Fig. 6:**
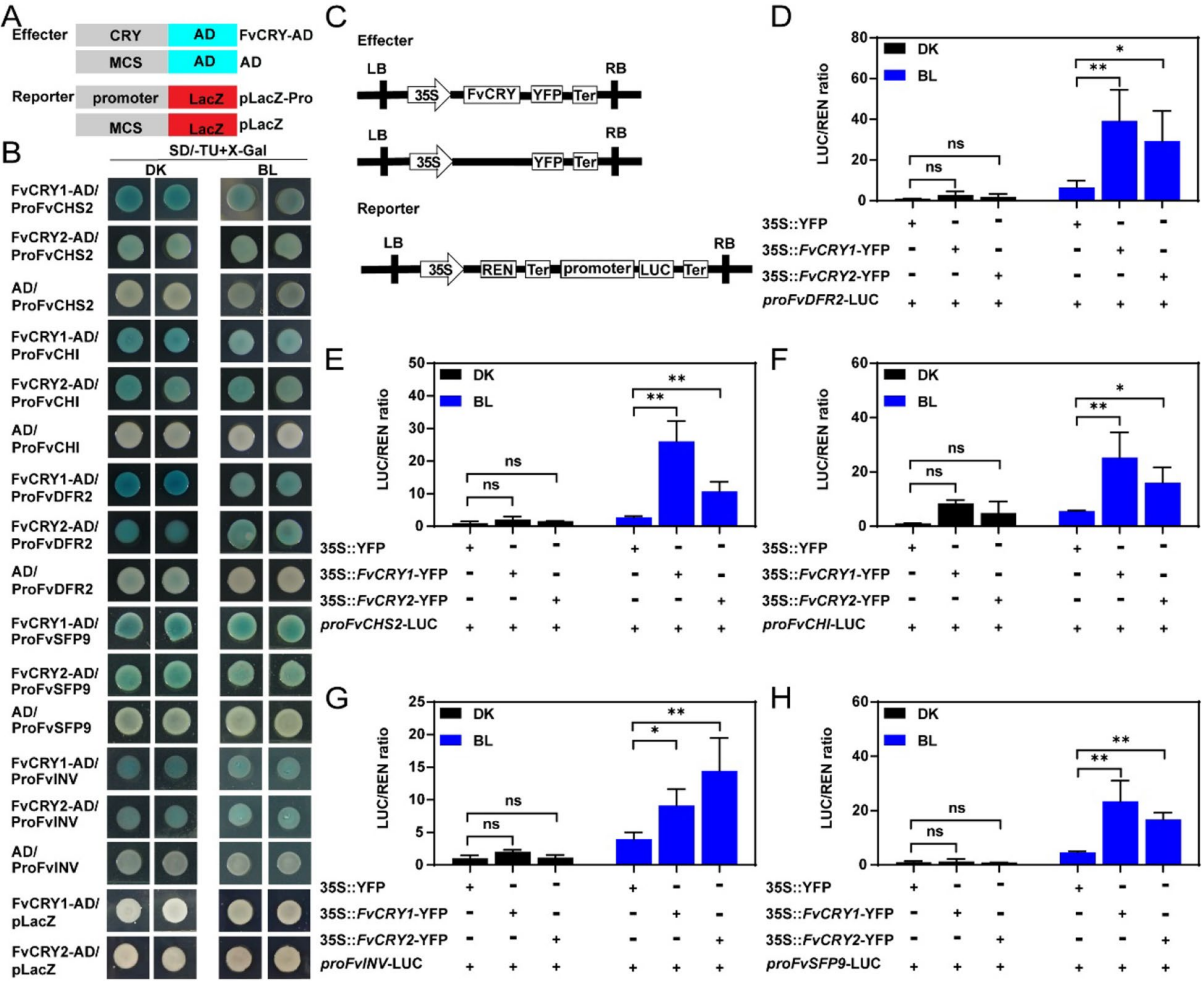
FvCRYs can activate the expression of target genes. **A** Schematic diagram of the vectors used in the yeast one-hybrid experiments. **B** Yeast one-hybrid assay showing FvCRYs bind to the promoter of genes related to anthocyanin and sugar accumulation. Blue color indicates the expression of the LacZ reporter. **C** Schematic diagram of the vectors used in the Dual-LUC experiments. **D**-**H** The relative quantification of LUC activity for the Dual-LUC assay. The LUC/REN ratio of control (*35S*::YFP/promoter, DK) was set to 1. Asterisks indicate a significant difference relative to the control (*, *P* < 0.05, **, *P* < 0.01, ns means no significance, ANOVA). Data are means ± SD from three independent biological replicates. DK: Dark; BL: Blue Light

FvCRYs are known to promote the expression of several key genes, including anthocyanin synthesis-related genes (*FvDFR2*, *FvCHI*, *FvCHS2*) and sugar metabolism and transporter genes (*FvINV* and *FvSFP9*). Moreover, promoter analysis revealed that the promoters of these genes all contain E-box cis-elements (Fig. S5). Based on these findings, we hypothesize that FvCRYs may directly regulate anthocyanin and sugar accumulation in fruit by enhancing the expression of these genes. Yeast one-hybrid experiments showed that FvCRY1 and FvCRY2 do bind to the promoters of these genes related to anthocyanin and sugar accumulation (Fig. 6B). Furthermore, Dual-LUC assay results showed that the activity of *LUC* driven by the promoters of *FvCHI*, *FvCHS2*, *FvDFR2*, *FvINV*, and *FvSFP9* was significantly enhanced when co-expressing FvCRYs under blue light (Figs. 6D-H), implying that blue light may directly regulate the accumulation of anthocyanins and sugars by activating FvCRYs.

## Discussions

Cryptochromes (CRYs) are a crucial class of blue light receptors first discovered in *Arabidopsis thaliana* and widely distributed across plants, animals and microorganisms. In plants, CRYs primarily function to sense external light environments, especially blue light and UV-A signals. Structurally, the N-terminus of CRYs shares high homology with bacterial DNA photolyases (Mei and Dvornyk 2015). Notably, UV light induces the formation of cyclobutane pyrimidine dimers (CPDs) in DNA, which photolyases can bind without Light. However, photolyase activation and dimer breakdown require photons within the 350 and 450 nm range. This repair process releases the photolyase from the DNA strand, restoring normal structure of DNA strand (Christou et al. 2023). Thus, while photolyase can bind DNA directly in a light-independen manner, its catalytic repair function is light-dependent.

Several studies in both plants and animals suggest that CRYs may directly bind DNA to regulate transcription. In mouse liver, Koike performed chromatin immunoprecipitation sequencing (ChIP-seq) of seven core factors in the circadian clock pathway, including CRY1 and CRY2. They found that CRY1 recognizes over 16,000 genomic loci, with nearly one-third unique to CRY1, and more than 10,000 sites are recognized by CRY2, with one-fifth unique to CRY2. This demonstrates that animal CRYs can recognize numerous DNA loci on a genome-wide scale. By analyzing the sequence features of these loci, they found that the E-box sequence is the main motif recognized by CRYs (Koike et al. 2012). Similarly, in *Arabidopsis*, ChIP-seq identified 6249 AtCRY2 target genes with enriched E-box cis-elements. However, it was not demonstrated in any of these studies whether CRYs directly binds the E-box motif. In our study, we used yeast one-hybrid assays to confirm that the strawberry blue light receptors FvCRY1 and FvCRY2 can directly bind the E-box motif (Fig. 5). Notably, this binding occurred in the dark, consistent with findings by Yang that dark-treated AtCRY2 could bind the G fragment of the *FT* gene (Yang et al., 2018b). However, unlike their study, we did not observe blue light enhancement of this binding, likely due to differences in experimental systems. We were using a yeast one-hybrid system, whereas they expressed the AtCRY2 protein in mammalian cells (HEK293T).

**Fig. 7:**
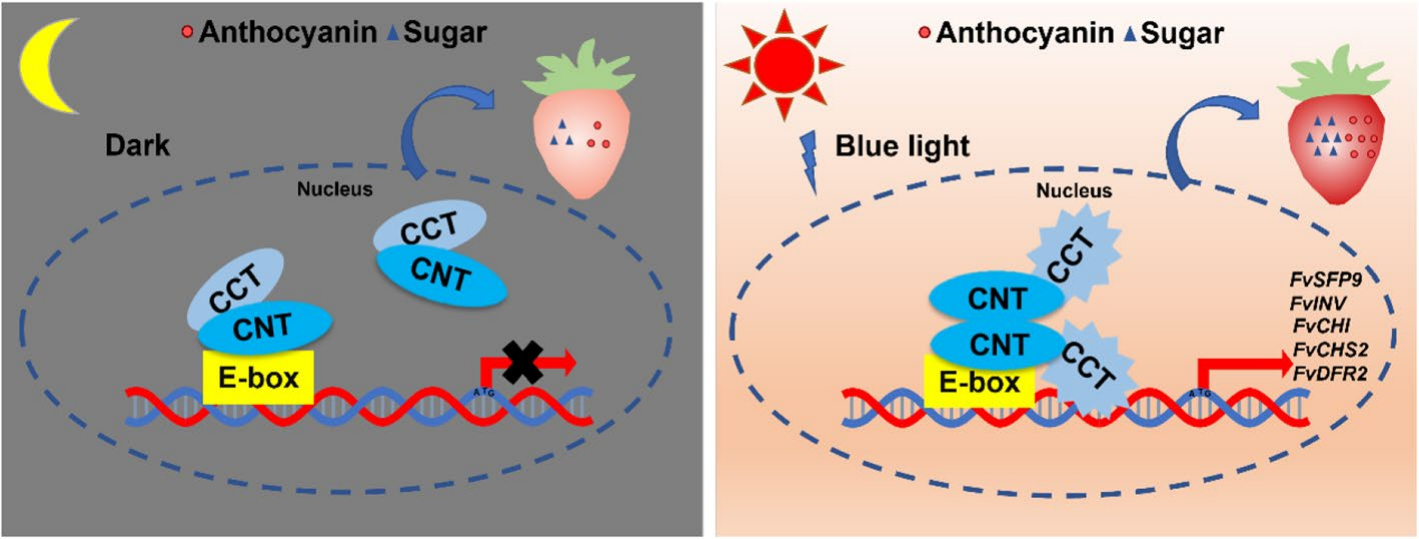
A model for the regulation of strawberry anthocyanin and sugar metabolism by FvCRYs. In the dark, FvCRYs bind downstream target genes but do not activate their transcription. After exposure to blue light, FvCRYs were activated to promote the transcription of downstream target genes involved in anthocyanin and sugar metabolism, thereby increasing the anthocyanin and sugar content of strawberries

In this study, we found a significant increase in both anthocyanin and sugar content in transgenic strawberry fruits overexpressing either FvCRY1 or FvCRY2 (Fig. 3), which coincided with elevated expression of genes related to anthocyanin and sugar accumulation in transgenic strawberry fruits (Figs. 3E, F). By analyzing the promoter regions of these genes, we found that these genes contain E-box cis-elements (Fig. S5), and yeast one-hybrid assays confirmed that FvCRYs binding to these promoters is light-independent (Fig. 6B). Under blue light, FvCRY1 and FvCRY2 enhanced *E-box*-driven expression of *LUC* genes (Figs. 5D, F), consistent with findings by Yang (Yang et al., 2018a)that the active form of AtCRY2 promotes *LUC* expression driven by the *FT* gene's G fragment. Previous studies have focused on the indirect effects of CRYs on downstream gene expression through protein–protein interactions. In contrast, our study demonstrates that FvCRYs directly regulate gene expression by binding promoter regions, thereby influencing fruit quality (Fig. 7). This expands our understanding of how CRYs mediate light signaling to regulate plant growth and development.

Transcription factors generally have two typical domains: the DNA-binding domain (DBD) and the transcriptional activation domain (AD). The functions of these two domains are relatively independent. In our study of the transcriptional activity of FvCRYs, we observed that the N-terminus alone does not have transcriptional activation activity. However, when the N-terminus was extended to include the NC80 domain, the truncated form of FvCRYs exhibited activation activity comparable to that of the full-length protein (Fig. 4). This implies that the C-terminus is important for the activation function of FvCRYs. Notably, testing the C-terminus in isolation revealed no self-activating activity (Fig. S6). Previous studies have demonstrated that dimerization is essential for the function of CRY proteins. For instance, GUS-CCT, which forms constitutive dimers, induces a constitutive photomorphogenic phenotype in transgenic *Arabidopsis* (Yang et al. 2000). We also tested the self-activating activity of GUS-FvCCT and found that it lacked such activity (Fig. S6). This finding implies the conformation of the full-length FvCRYs protein appears to be critical for its activation ability, as observed in studies of AtCRY2's activation of the G fragment of the *FT* gene.

From an evolutionary perspective, the N-terminus of CRY proteins is relatively conserved, whereas their C-termini exhibit variable lengths and lower conservation both between and within species (Figs. S1, S2). Structurally, the C-terminus of FvCRYs often appears as an intrinsically disordered region (IDR). Intriguingly, the transcriptional activation domains of many transcription factors also exhibit disordered properties, similar to the IDRs of FvCRYs. These disordered regions can adopt highly flexible and dynamic conformations, allowing them to adapt to various environmental conditions. This flexibility likely explains the extensive involvement of plant CRY proteins in multiple growth and developmental processes. Additionally, we observed that under low screening pressure (SD/-TH), the full-length FvCRY1 protein exhibited weak self-activating activity in the dark (Fig. 4B). Given that the dark function of AtCRY2 has recently been confirmed (Zeng et al. 2025), the self-activating activity of FvCRY1 in the dark may also suggest a potential function under dark conditions. Further investigation is warranted to elucidate the specific roles of FvCRY1 in different light environments.

## Materials and methods

### Plant material and cultivation conditions

Plant materials used in this study included octoploid cultivated strawberry (*Fragaria* × *ananassa*) variety ‘Ningyu’ for genetic transformation, tobacco (*Nicotiana benthamiana*) for dual-luciferase experiments, and *Arabidopsis thaliana* Columbia (Col) ecotype, *cry1* and *cry1 cry2* mutants for functional analysis of FvCRY1 and FvCRY2. All the plants were cultivated in a greenhouse with a Light intensity of 100–150 μmol m^−2^ s^−1^, a temperature of 22 °C, a photoperiod of 16 h of Light and 8 h of darkness, and a relative humidity of 70%.

### RNA extraction and real-time quantitative PCR

The fruit samples were ground into powder in liquid nitrogen, and total RNA was extracted with an RNA extraction kit (Cat. DP441, Tiangen, Beijing, China). Subsequently, cDNA was generated by using a reverse transcription kit (AE311-02, Transgenics, Beijing, China). The RT-qPCR system and data analysis were performed as previously described (Xu et al. 2023). In this study, gene-specific primers were designed via NCBI Primer-BLAST and validated against the whole strawberry genome by BLAST. The primers utilized are listed in the Supplementary Table S1.

### Phylogenetic and expression pattern analysis

The amino acid sequences of woodland strawberry FvCRY1 and FvCRY2 were obtained from the Genome Database of Phytozome 13 (https://phytozome-next.jgi.doe.gov/). Other homologous proteins of FvCRYs from different species were obtained from the National Center for Biotechnology Information (NCBI) database (http://www.ncbi.nlm.nih.gov/). Multiprotein sequence comparison was performed using DNAMAN and ESPript (Robert and Gouet 2014) with default parameters. The phylogenetic evolutionary tree was constructed by neighbor-joining method with 1000 bootstrap replicates using MEGA 11. The expression levels of FvCRYs in various stages of woodland strawberry development were obtained from the eFP (electronic Fluorescent Pictograph) browser (https://bar.utoronto.ca/efp_strawberry/cgi-bin/efpWeb.cgi).

### Subcellular localization assay

The CDS of *FvCRYs* was inserted into the pHB-YFP to construct FvCRYs-YFP fusion expression vectors. The vector was transferred into *Agrobacterium tumefaciens* GV3101, and fluorescence signals were observed in tobacco leaves injected by a confocal laser scanning microscope (Leica TCS SP5II), with empty pHB-YFP serving as a control. The primers used are listed in the Supplementary Table S2.

### Determination of anthocyanin content

Determination of total anthocyanins was carried out as previously reported (Li et al. 2020). The fruit samples frozen in Liquid nitrogen were ground into powder. 0.1 g of powder was mixed with 600 μL of methanol-diluted 1% HCl (v/v) and incubated at 4 °C overnight. 400 μL ddH_2_O was added, centrifuged and the supernatant was collected. 1 mL chloroform was mixed with supernatant. After centrifugation, take the upper aqueous phase to measure the absorbance values at 530 nm and 657 nm. The anthocyanin content was calculated as (A530-A657)/FW. FW refers to the fresh weight of the sample and all experiments were performed with three replicates.

### Determination of soluble solids and sugar fractions

The soluble solids content was determined by a saccharimeter (LB20T, SWEVY, Guangzhou, China). The content of sugar was measured via high-performance liquid chromatography (HPLC) as follows: standard solutions of glucose, fructose, and sucrose were prepared by diluting each to a concentration of 100 ng/mL in ultrapure water. These standard solutions were then subjected to gradient dilution, and the resulting solution were analyzed by HPLC to generate standard curves, with sugar content plotted on the x-axis and the peak area on the y-axis.

The strawberry fruit samples were ground into powder in Liquid nitrogen. 3.0 g powder was mixed with 6 mL of deionized water, and ultrasonicated for 15 min. The mixture was then centrifuged at 10,000 rpm for 15 min, and the supernatant was filtered through a 0.22 um filter membrane for HPLC analysis.

The HPLC used for the analysis included an LC3000 high performance liquid chromatograph (CXTH, Beijing, China) equipped with a differential detector (KNAUER, Berlin, Germany) and a ZORBAX NH2 column (4.6X250 mm, 5 um) (Agilent Technologies, CA, USA). The mobile phase consisted of acetonitrile: water (75:25) at a flow rate of 0.6 mL/min, with the column temperature maintained at 25 °C. The above assays were replicated three times.

### Yeast one-hybrid assay

Yeast one-hybrid assays were performed as described previously (Li et al. 2010). The full-length coding sequences of and N-terminal/C-terminal of *FvCRY1* and *FvCRY2* were cloned into the the yeast expression vector mpB42AD vector to generate the constructs mpB42AD-FvCRY, mpB42AD-FvCNT, mpB42AD-FvCCT, respectively. Additionally, approximately 2 kb promoter fragments upstream of the start codon (ATG) of each candidate gene were cloned into the reporter vectors pLacZ. The resulting expression and reporter vectors were co-transformed into the yeast strain EGY48. The empty vector (pB42AD or pLacZ) was used as a negative control. The primers used for constructing expression and reporter vectors are listed in the Supplemental Table S3.

### Dual-luciferase assay

The expression vectors pHB-FvCRY1-YFP and pHB-FvCRY2-YFP driven by *35S* promoter were transformed into *A. tumefaciens* strain GV3101. An approximately 2-kb fragment upstream of the start codon of the target gene was amplified and cloned into the pGreenII0800-LUC vector. The resulting pGreenII0800-promoter vectors were transformed into *A. tumefaciens* strain GV3101 (harboring pSoup-p19). The reporter strain was then mixed with effector strains carrying either pHB-FvCRY1-YFP or pHB-FvCRY2-YFP, or the negative control pHB-YFP, and co-infiltrated into tobacco leaves. Infected plants were kept in the dark for 12 h and then transferred to Light for 36 h, and leaves were collected after 36 h of exposure to light for dual luciferase assays. The Dual Luciferase Reporter Gene Assay Kit (11402ES60, Yeasen, Shanghai, China) was used for these assays. For the blue Light treatment, the injected tobacco was kept in darkness for 12 h and then transferred to 30 μmol m^−2^ s^−1^ blue Light or dark for 36 h. All the experiments were replicated three times. The primers used for the Dual-LUC assay are listed in the Supplemental Table S4.

### Transcriptional activity assay

According to their domain structures, the coding sequences of *FvCRY1* and *FvCRY2* were truncated into specific fragments: for FvCRY1, the fragments were 2022 bp, 1827 bp and 1449 bp; for *FvCRY2*, the fragments were 1938 bp, 1740 bp and 1446 bp. These fragments correspond to the full-length protein, full-length deletion CT and N-terminal domain, respectively. Each truncated sequence was inserted to yeast expression vector mpGBKT7 (BD domain was infused with C terminus of target protein) and transformed into yeast strain AH109. The self-activating activity of different truncated forms of FvCRY1/FvCRY2 proteins was determined by observing the growth of yeast cells on defective media (SD/-TRP, SD/-TRP/-HIS, SD/-TRP/-HIS/-ADE) under blue light (30 μmol m^−2^ s^−1^) or darkness.

Additionally, a reporter vector for *LUC* expression driven by the GAL4 upstream activating sequence (UAS) and TATA box was constructed. Effector vectors harboring fusions of FvCRY1/FvCRY2 fragments with the GAL4 DNA-binding domain was also constructed. These reporter and effector vectors were separately transformed into *A. tumefaciens* strain GV3101 and co-infiltrated into tobacco leaves. The transcriptional activation activity of different FvCRYs fragments was evaluated using the LUC/REN luciferase assay. All the experiments were replicated three times. The primers used for the transcriptional activity assay are listed in the Supplemental Table S5.

### Stable genetic transformation of *Arabidopsis* and Strawberry

The full-length coding sequences (CDS) of *FvCRY1* and *FvCRY2* were cloned into the pHB-YFP vectors to generate pHB-FvCRY1-YFP and pHB-FvCRY2-YFP coustructs for plant expression. These constructs were introduced into the *A. tumefaciens* strain GV3101 and then transformed into *Arabidopsis* by floral dip method (Clough and Bent 1998). Transgenic lines overexpressing *FvCRY1-YFP* in the *cry1* mutant (*FvCRY1*-OE/*cry1*) and *FvCRY2-YFP* in the *cry1 cry2* double mutant (*FvCRY2-*OE*/cry1 cry2*) were screened on Murashige–Skoog (MS) medium supplemented with 50 mg/L Hyg. Homozygous T2 generation plants were used for phenotyping. Primers for plasmid construction are listed in the Supplementary Table S6.

For transformation into cultivated strawberry (*Fragaria* × *ananassa*) cv. Ningyu, *A. tumefaciens* GV3101 harboring pHB-FvCRY1-YFP or pHB-FvCRY2-YFP was used and the detailed transformation methods were performed as described previously (Gao et al. 2020).

### Western blot

Western blot analysis was performed as previously described (Li et al. 2024).To examine the protein expression levels in transgenic plant of *FvCRY1*-OE/*cry1* and *FvCRY2*-OE/*cry1 cry2*. Antibodies against YFP (Sigma-Aldrich, MO, USA) and Actin (Abmart, Shanghai, China) were used for detection. Wild-type (Col-0), *cry1*, and *cry1 cry2* mutant lines were included as controls.

### Data analysis

Data are presented as the mean ± standard deviation (SD) of at least three independent experiments or three biological replicates. Statistical comparisons between experimental and control groups were determined by Student’s *t*-tests. For analysis involving multiple experimental groups, analysis of variance (ANOVA) was conducted using GraphPad Prism version9.5, followed by Tukey's multiple comparisons test where appropriate.

## Supplementary Information

Supplementary Material 1.

Supplementary Material 2.

Supplementary Material 3.

Supplementary Material 4.

Supplementary Material 5.

Supplementary Material 6.

Supplementary Material 7.

## Data Availability

The data will be available from the corresponding author upon reasonable request.

## Abbreviations

WT: Wild-type
RT-qPCR: Reverse transcription-quantitative polymerase chain reaction
35S: Cauliflower mosaic virus promoter
Hyg: Hygromycin B
X-Gal: 5-Bromo-4-chloro-3-indoxyl-β-D galactopyranoside
PAL: Phenylalanine ammonia lyase
ANR: Anthocyanidin reductase
UFGT: UDP-glucose flavonoid 3-O-glucosyltransferase
CHS: Chalcone synthase
CHI: Chalcone isomerase
F3H: Flavanone-3-hydroxylase
DFR: Dihydroflavonol-4-reductase
ANS: Anthocyanidin synthase
SUT: Sucrose transporter
MST: Monosaccharide transporter
SWEET: Sugars will eventually be exported transporter
SFP: Sugar facilitator protein
INV: Invertase
SUS: Sucrose synthase
SPS: Sucrose phosphate synthase
GUS: β-Glucuronidase
LUC: Luciferase
REN: Renilla Luciferase
