# Supplementary material for: Strawberry cryptochrome FvCRY1 and FvCRY2 transcriptionally regulate anthocyanin biosynthesis and sugar metabolism
Source: Mol Hortic. 2025 Dec 2;5:63. doi: 10.1186/s43897-025-00197-5 (PMC12670867; doi:10.1186/s43897-025-00197-5)
Supplement: Supplementary file 7 — Supplementary Material 7. [file 43897_2025_197_MOESM7_ESM.pdf]

A

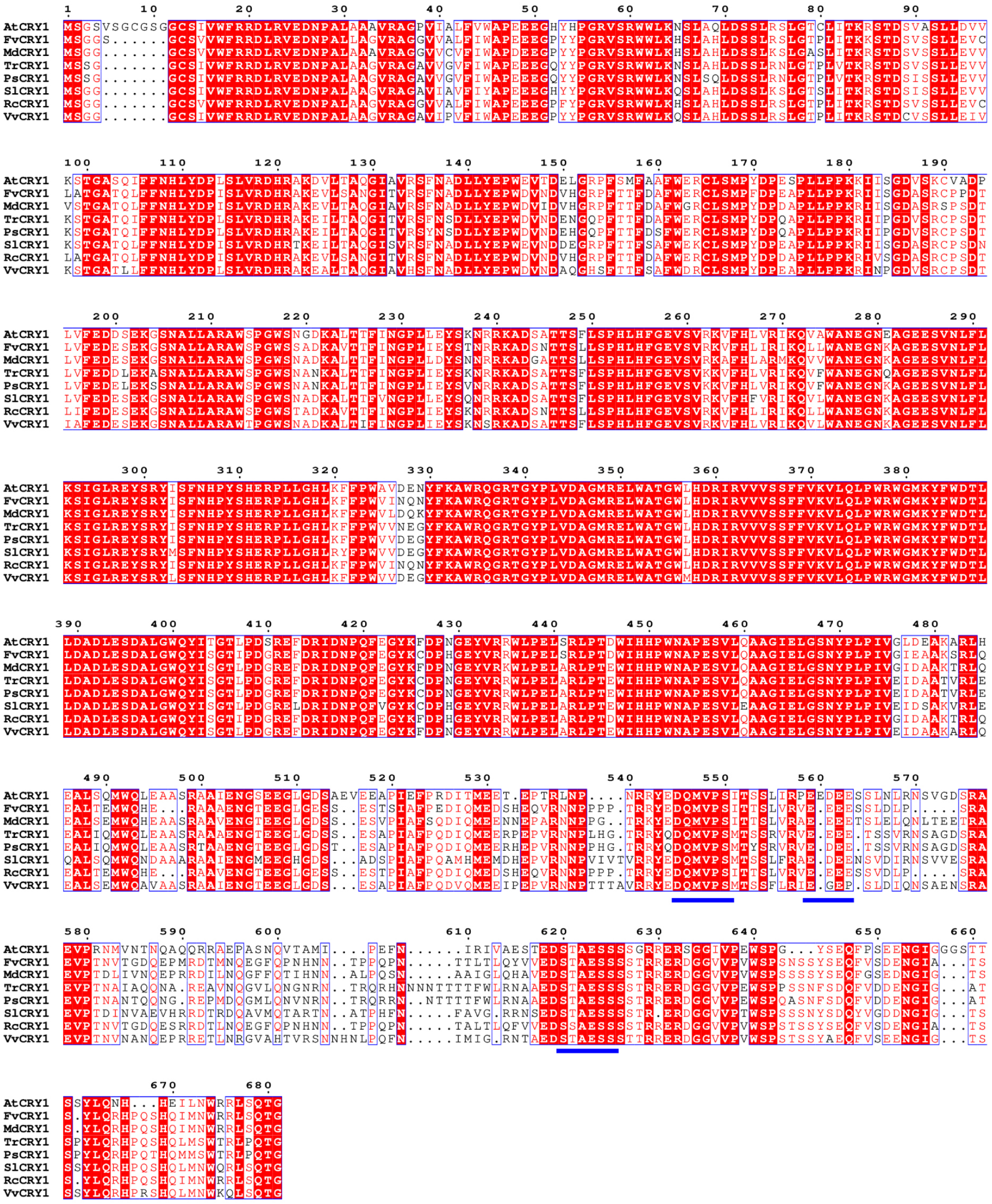

Fig. S1 Comparison of multiple protein sequences of FvCRY1 and CRY1 from other species. The blue horizontal lines indicate DAS structural domains. At, *Arabidopsis thaliana*. Fv, *Fragaria vesca*. Md, *Malus domestica*. Ps, *Pisum sativum*. Sl, *Solanum lycopersicum*. Vv, *Vitis vinifera*. Rc, *Rosa chinensis*. Tr, *Trifolium repens.*

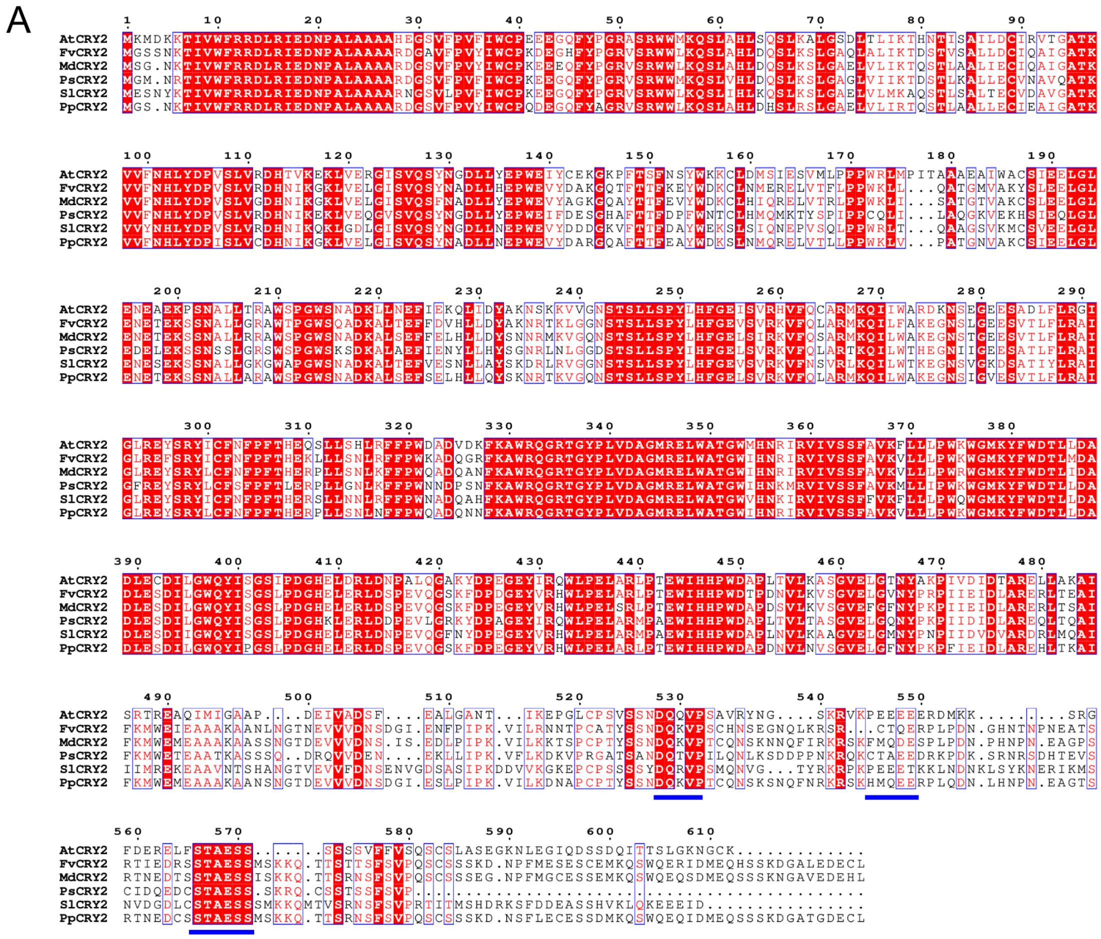

Fig. S2 Comparison of multiple protein sequences of FvCRY2 and CRY2 from other species. The blue horizontal lines indicate DAS structural domains. At, *Arabidopsis thaliana.* Fv, *Fragaria vesca*. Md, *Malus domestica*. Ps, *Pisum sativum*. Sl, *Solanum lycopersicum.* Pp, *Prunus persica.*

FvCRYs.

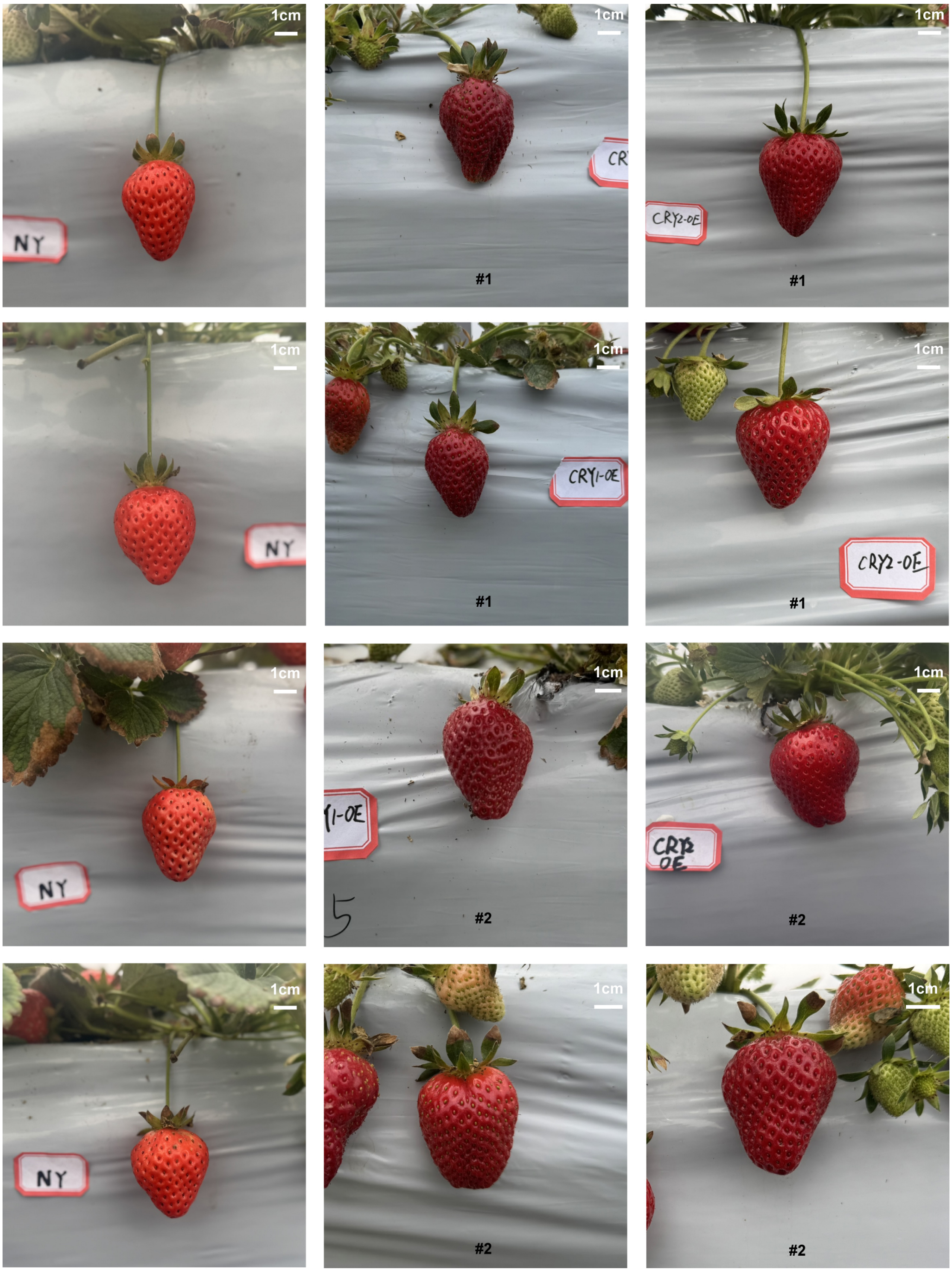

NY | 35S::*FvCRY1*-YFP | 35S::*FvCRY2*-YFP

Fig. S3 The fruit color phenotypes of transgenic strawberries with overexpression of FvCRYs.

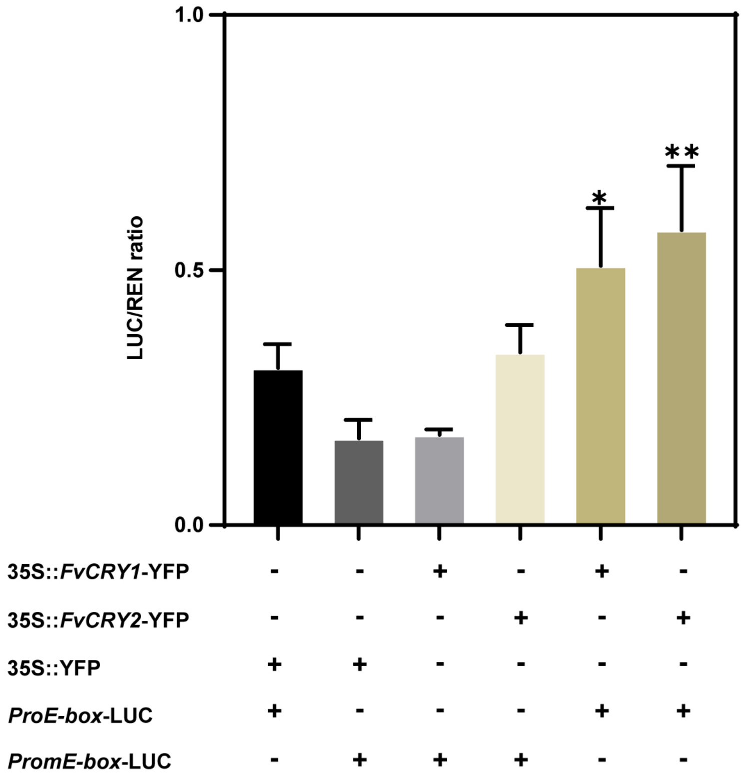

Fig. S4 Dual luciferase assay for activation of E-box and mE-box by FvCRYs. Asterisks indicate a significant difference relative to the control (*P < 0.05, **P < 0.01, Student's t-test). Data are means ± SD from three independent biological replicates.

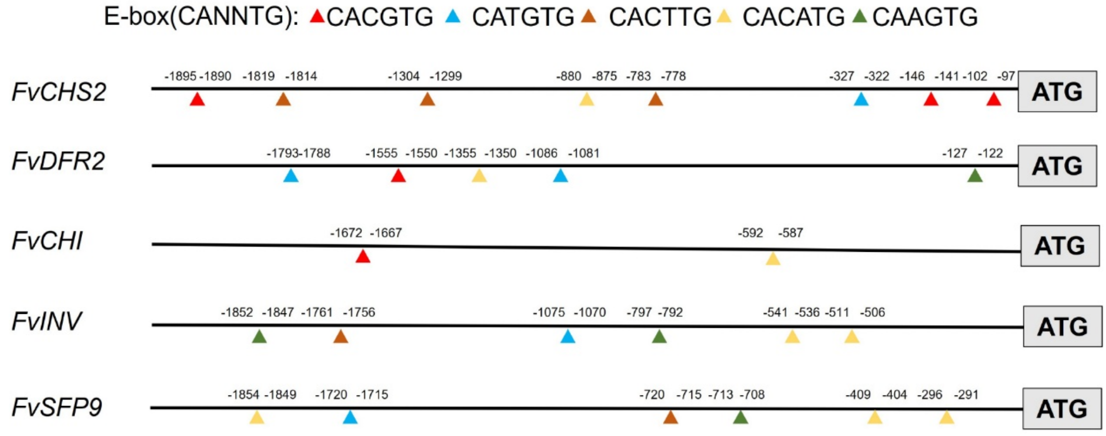

Fig. S5 Analysis of E-box cis-elements on promoters of genes related to anthocyanin and sugar metabolism.

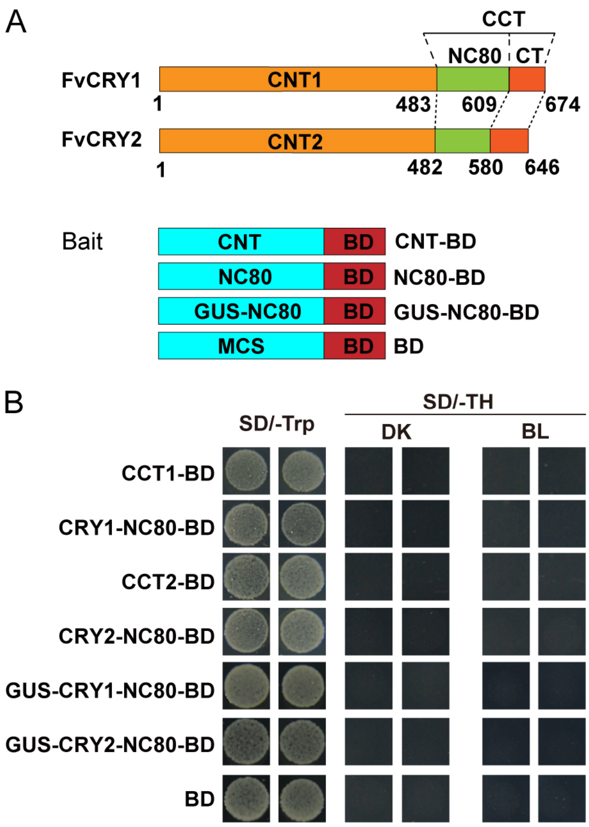

Fig. S6 Analysis of the individual C-termini of FvCRYs and GUS-CCT transcriptional activation activity. (A) A schematic representation of the different truncated forms of FvCRYs. (B) Self-activating activity of FvCRYs in yeast cells. SD-T, SD-TH, SD-THA stands for histidine(H), adenine(A), and tryptophan(T) defective media.
